# Unraveling hair graying: insights into melanocyte biology and the aging process

**DOI:** 10.3389/fcell.2026.1815236

**Published:** 2026-10-09

**Authors:** Siang-Yu Chen, Hao-Jui Weng, Yu-Wen Yeh, Chung-Ping Liao

**Affiliations:** 1 School of Medicine, College of Medicine, Taipei Medical University, Taipei, Taiwan; 2 Far Eastern Memorial Hospital, New Taipei City, Taiwan; 3 Graduate Institute of Clinical Medicine, College of Medicine, Taipei Medical University, Taipei, Taiwan; 4 Department of Dermatology, Taipei Medical University-Shuang Ho Hospital, New Taipei City, Taiwan; 5 Department of Dermatology, School of Medicine, College of Medicine, Taipei Medical University, Taipei, Taiwan; 6 International Ph.D. Program in Cell Therapy and Regenerative Medicine, College of Medicine, Taipei Medical University, Taipei, Taiwan; 7 Division of Dermatology, Tri-Service General Hospital Songshan Branch, National Defense Medical University, Taipei, Taiwan; 8 Department of Dermatology, School of Medicine, National Defense Medical University, Taipei, Taiwan; 9 Graduate Institute of Medical Sciences, National Defense Medical University, Taipei, Taiwan; 10 Graduate Institute of Medical Sciences, College of Medicine, Taipei Medical University, Taipei, Taiwan; 11 Center for Precision Medicine and Translational Cancer Research, Taipei Medical University Hospital, Taipei, Taiwan; 12 Cell Physiology and Molecular Image Research Center, Wan Fang Hospital, Taipei Medical University, Taipei, Taiwan

**Keywords:** aging, hair graying, melanocyte, melanogenesis, pigmentation

## Abstract

Melanogenesis and pigmentation are crucial processes for normal skin and hair, and they are regulated by complex factors. Unlike epidermal pigmentation, follicular melanogenesis depends on cyclic replenishment from a bulge melanocyte stem cell reservoir, making its functional decline generally irreversible yet clinically reversible under rare conditions. Herein, we systematically review the core biological processes governing follicular pigmentation, including embryonic specification, niche homing, cyclic lineage migration, and microenvironmental signaling networks. We further integrate emerging single-cell and spatial omics findings to propose a novel, three-stage chronological model of canities, bridging mouse-derived mechanisms with human clinical evidence. There are multiple conditions that lead to hair graying, such as aging, premature hair graying, the stem cell niche environment, and the biological variety of melanocytes and hair follicles, with aging being the most common factor among these. In brief, the exact mechanisms of hair pigmentation and depigmentation remain elusive. Herein, we review the physiology and pathology of hair pigmentation and depigmentation, and summarize the mechanisms that might be affected during human hair graying.

## 1 Introduction

Hair is a conspicuous mammalian trait, and it can be expressed in distinct colors (Caro and Mallarino, 2020). In humans, hair graying is one of the hallmarks of human aging; however, the exact and detailed mechanisms of hair graying remain unclear (O'Sullivan et al., 2021). In general, aging-related hair graying is a non-reversible physiological alteration, but clinical evidence has demonstrated sporadic cases of hair repigmentation under certain medications or disease conditions due to unknown mechanisms (Yale et al., 2020; Chan et al., 2019; Rivera et al., 2017; Shaffrali et al., 2002), which emphasizes our lack of understanding of the physiology of hair pigmentation and depigmentation.

Hair functions in protection, sensation, thermoregulation, and social communication (Schneider et al., 2009). Healthy hair in both men and women represents youth and vitality. The appearance of hair can vary in different human subgroups and ages. Unlike skin pigmentation, which relies on melanins, hemoglobins, and carotenoids (Tobin et al., 2005), hair pigmentation solely depends on the presence of distinct melanins and their densities. These distinct human hair colors are primarily dictated by the specific ratio and absolute density of brownish-black eumelanin and reddish-yellow pheomelanin (Ito and Wakamatsu, 2003), both of which are regulated through the classic enzymatic cascades of the melanogenesis pathway (Figure 1).

**FIGURE 1:**
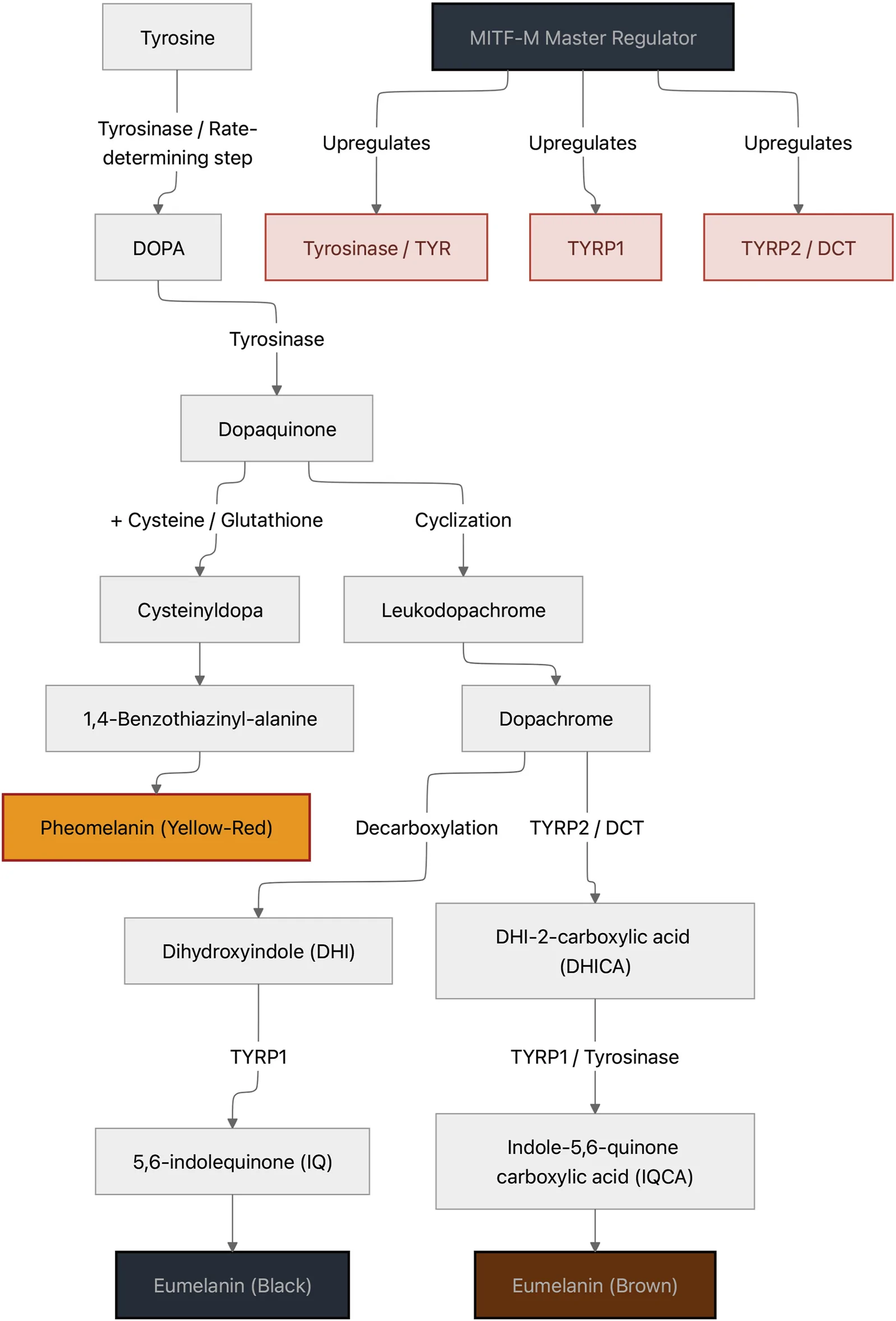
Biochemical signaling and enzymatic cascades of melanin biosynthesis in follicular melanocytes. This schematic flowchart illustrates the synchronized enzymatic pathways governing melanogenesis within the hair follicle architecture, controlled upstream by the transcription factor MITF. The pathway initiates with the conversion of tyrosine to dopaquinone, a rate-determining step catalyzed by tyrosinase (TYR). Arrows delineate the subsequent divergence into primary pigment branches based on substrate availability. The leftward branch demonstrates the reaction of dopaquinone with cysteine or glutathione to synthesize pheomelanin (yellow-red) *via* a cysteinyldopa intermediate. Conversely, the rightward branch charts the autonomous cyclization of dopaquinone into dopachrome, which further bifurcates into the DHI-derived black eumelanin pathway mediated by TYRP1, and the DHICA-derived brown eumelanin pathway sequentially regulated by TYRP2/DCT and TYRP1. Black block arrows denote transcriptional upregulation of these core melanogenic enzymes by MITF. Molecular pathways are synthesized based on core mechanisms reviewed in (Ito and Wakamatsu, 2003; D'Alba and Shawkey, 2019; Fukuda et al., 2021).

In this review article, we discuss aspects of the melanogenesis pathway, melanocyte developmental biology, molecules and signaling pathways regulating melanogenesis and melanocytes, and possible mechanisms associated with repigmentation.

## 2 The biological development of melanocytes

### 2.1 Neural crest origins and lineage specification

Melanocytes originate from neural crest cells (NCCs), a transient, multipotent embryonic cell population arising between the neural plate and non-neural ectoderm (Dawes and Kelsh, 2021; Petit et al., 2016). Originating from the dorsal-most part of the neural tube, NCCs experience a serial process of transition, migration, differentiation, and proliferation (Glover et al., 2015). In the melanocyte lineage, melanoblast-glial progenitor cells, melanoblasts, and functional melanocytes appear in sequential order, regulated by embryonic signaling molecules including WNT, bone morphogenetic protein (BMP), endothelin 3 (EDN3), and stem cell factor (SCF) (Mica et al., 2013; Peters et al., 2002).

### 2.2 Melanoblast migration and bulge niche homing

Melanoblasts migrate along the dorsolateral pathway to their destination in the epidermis (Glover et al., 2015). In addition, Schwann cell precursors migrating along the ventral pathway can also differentiate into melanocytes (Saleem, 2019; Bonaventure et al., 2013; Sommer, 2011). During embryonic development, melanoblasts from both migratory origins converge at specific niches, such as the hair follicle bulge, where they establish the initial pool of melanocyte stem cells (Nishimura, 2011). This developmental homing marks the transition of lineage cells from an actively migrating embryonic state to a restricted, quiescent stem cell population geared for lifelong tissue homeostasis (Nishimura, 2011) (Figure 2).

**FIGURE 2:**
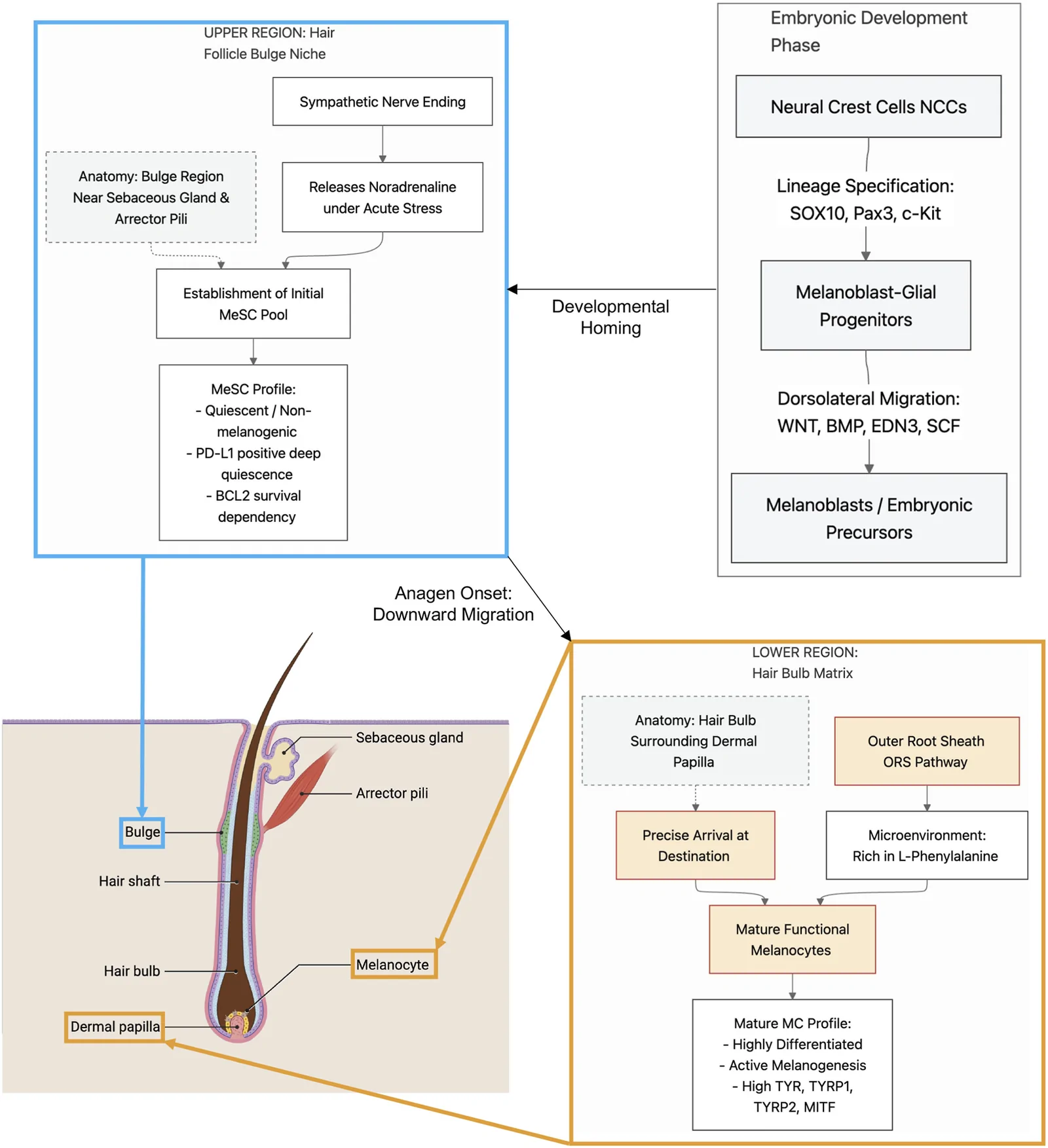
Spatiotemporal mapping of melanocyte stem cell (MeSC) embryonic development, niche homing, and adult lineage profiles. This multi-panel diagram contrasts the embryonic lineage progression of the melanocytic system with its localized spatiotemporal topography during adult tissue homeostasis. The upper sequential flow delineates the transition of neural crest cells (NCCs) into migratory melanoblasts and their subsequent developmental homing into the initial hair follicle bulge reservoir. The lower compartmentalized follicle model highlights the distinct cellular profiles and markers defining different anatomical domains. The upper bulge region depicts the quiescent, non-melanogenic MeSC pool, illustrating its maintenance under PD-L1 control and its structural innervation by sympathetic nerve endings that release noradrenaline. The downward dashed arrow represents the functional reactivation and step-wise differentiation of progenitor cells migrating along the outer root sheath (ORS) pathway. This lineage transit culminates in the lower hair bulb matrix, where mature functional melanocytes exhibit high melanogenic capacity within an L-phenylalanine-rich microenvironment. Developmental and homeostatic models are constructed based on (Peters et al., 2002; Nishimura, 2011; Zhang et al., 2020). The diagram was created with BioRender.com.

### 2.3 Melanocyte stem cells in the hair bulge niche

Melanocyte stem cells (MeSCs) are established during embryogenesis when neural crest-derived melanoblasts migrate into the developing hair follicle bulge, a localized recruitment process tightly regulated by the same embryonic signaling cascade including WNT and SCF pathways (Peters et al., 2002; Nishimura, 2011).

MeSCs are the cell-of-origin of melanocytes of hair follicles during homeostasis. During adult hair cycles, these embryonic pathways are reactivated to coordinate MeSC activation and differentiation (Nishimura, 2011). To sustain this pool, localized TGF-beta signaling maintains MeSC quiescence, whereas active Wnt signaling coordinates MeSC differentiation during hair cycle regeneration, ensuring that proper Wnt dosage preserves the stem cell reservoir against premature depletion and subsequent hair graying (Rabbani et al., 2011; Paus et al., 2024). MeSCs reside in the hair follicle bulge niche, and their activation, differentiation, and proliferation are associated with hair cycle changes (Glover et al., 2015; Nishimura, 2011; Qiu et al., 2019; Lang et al., 2013; Nishimura et al., 2002; O'Sullivan et al., 2021). In addition to maintaining melanocyte homeostasis, research evidence suggested that MeSCs can also contribute to wound healing during tissue repair and regeneration (Chou et al., 2013).

MeSCs are non-melanogenic, and they adhere to hair follicle stem cells in the bulge area of hair follicles (Nishimura et al., 2002). The bulge reservoir for MeSCs is critical for melanocyte activity. Hair graying becomes irreversible due to the loss of bulge MeSCs (Nishimura, 2011; Rabbani et al., 2011; Paus et al., 2024; Nishimura et al., 2005). MeSCs serve as the essential cellular reservoir that periodically replenishes differentiated melanocytes to reconstruct the functional hair follicle pigmentation unit (HFPU) in the hair bulb at the onset of each anagen phase (Nishimura, 2011; Tobin, 2011). However, the long-term maintenance of this bulge reservoir is vulnerable to niche disturbances, and its age-related loss leads to irreversible canities (Nishimura et al., 2005). Additionally, acute psychoemotional stress can dramatically accelerate the depletion of this MeSC reservoir; hyperactivation of sympathetic nerves triggers the local release of noradrenaline into the bulge niche, forcing quiescent MeSCs into rapid, uncoordinated proliferation and premature differentiation, which ultimately exhausts the stem cell pool and leads to irreversible hair graying (Lv et al., 2020).

Programmed death-ligand 1 (PD-L1) plays a novel and significant role in regulating the quiescent state and regenerative capacity of MeSCs. The PD-L1 gene is upregulated (5-fold higher) in quiescent MeSCs (qMeSCs) compared to proliferating melanoblasts. Functionally, PD-L1 restricts cell cycling and prevents cell death under quiescent conditions. The reversibility of hair graying is attributed to a subset of PD-L1–positive MeSCs that remain in a deeply quiescent state and resist depletion during aging (Palmer et al., 2024).

### 2.4 Melanocyte activation, differentiation, and migration along the hair follicle

At the onset of each anagen phase, MeSCs or their amplifying progeny initiate a downward migration from the bulge reservoir along the outer root sheath toward the hair bulb. During this transit, these cells undergo step-wise differentiation, transitioning from non-pigmented progenitors into fully mature, functional melanocytes precisely upon their arrival at the hair bulb matrix (Nishimura, 2011) (Figure 2). During transit, progenitor cells undergo step-wise differentiation into functional melanocytes that accumulate around the apex of the follicular papilla; once localized within this niche, they actively produce melanin for developing hair fibers (Tobin et al., 2005).

Notably, the anagen hair bulb houses distinct melanocyte subpopulations rather than a homogenous pool; these intra-bulbar cohorts exhibit significant heterogeneity, characterized by spatial variations in tyrosinase enzymatic activity, melanosome maturation stages (Stage III *versus* IV), c-Kit receptor expression levels, and distinct melanosome transfer capacities between melanocytes situated at the apex *versus* the upper outer regions of the hair papilla (Casalou et al., 2023). This highly active follicular melanogenesis near the anagen papilla is driven by a microenvironment rich in L-phenylalanine (Schallreuter et al., 1998) and is tightly synchronized with hair cycle progression, which spans anagen (growth), catagen (regression), and telogen (resting) phases (Oh et al., 2016).

As the hair follicle transitions into the catagen and telogen phases, this coupled pigmentation system undergoes cyclic involution. During catagen, melanogenesis is abruptly terminated, and mature bulbar melanocytes undergo programmed cell death or are cleared, while the upper reservoir remains protected to ensure a self-perpetuating system for subsequent cycles (Tobin, 2008; Tobin et al., 1999). In the early anagen of the next cycle, re-differentiating melanocytes are newly recruited from this upper reservoir (Tobin 2008). During the hair-graying process, the expression of MITF decreases, which impairs downstream melanogenesis and reduces melanosome transfer to matrix and precortical cells (Nishimura, 2011; Paus et al., 2024).

Chronologically, this cyclic behavior is orchestrated by sequential genetic cascades. Initially, SOX10 triggers the formation of melanoblast-glial bipotent progenitor cells (Domyan et al., 2019; Arora et al., 2021). Subsequently, MITF activation drives the expression of downstream markers such as TYRP2 and KIT (Goding and Arnheiter, 2019). These molecular pathways guide the precise segregation between quiescent MeSCs remaining in the bulge and the active, pigment-producing cohorts migrating into the hair bulb matrix (Mull et al., 2015).

## 3 Melanogenesis and pigmentation

### 3.1 Melanogenesis and pigment chemistry

Melanin is a biopolymer pigment synthesized within melanocytes (Mostert, 2021; Slominski et al., 2004). In mammals, two distinct types of melanin confer pigmentation to the skin, hair, and eyes: brownish-black eumelanin and reddish-yellow pheomelanin (Mostert, 2021; Slominski et al., 2004). Human hair color diversity is dictated by the absolute density and specific ratio of eumelanin to pheomelanin (Botchkarev, 2003). Black and brown hair feature a high density of eumelanin, blonde hair contains minimal overall pigment, and red hair exhibits a distinctly high proportion of pheomelanin (Ito et al., 2018; Ando et al., 2012). Chemical analyses demonstrate that eumelanin further diverges into DHI-derived black pigment and DHICA-derived brown pigment branches (Botchkarev, 2003; Bento-Lopes et al., 2023). DHICA-melanin exhibits superior hydroxyl radical scavenging activity compared to DHI-melanin (Ito et al., 2018; Serre et al., 2018). From an evolutionary perspective, localized genetic variation, such as a single amino acid substitution in TYRP1 driving Melanesian blonde hair, highlights the independent genetic trajectories shaping human pigmentation (Wang et al., 2023; Iida et al., 2020; Kenny et al., 2012). In non-pigmented hair, genes regulating melanin synthesis and melanocyte development, including Pax3, Sox10, c-Kit, Plexin C1, Melan-A, Pmel17, and MET, show marked downregulation.

Melanogenesis takes place within specialized lysosome-related organelles termed melanosomes (D'Alba and Shawkey, 2019). Microphthalmia-associated transcription factor (MITF) serves as the master transcriptional regulator of this enzymatic cascade (Kawakami and Fisher, 2017). MITF upregulates tyrosinase (TYR), tyrosinase-related protein 1 (TYRP1), and dopachrome tautomerase (DCT/TYRP2) (D'Mello et al., 2016). Rather than executing direct catalytic activation, TYRP1 and DCT physically interact with TYR to form a membrane-bound multi-enzyme complex that stabilizes TYR, protects it from premature degradation, and optimizes pigment output (Wang et al., 2024; Orlow et al., 1994; Kobayashi et al., 1998). TYR controls the rate-determining step by oxidizing tyrosine to dopaquinone, which rapidly converts to leucodopachrome and downstream substrates for eumelanin or reacts with cysteine/glutathione to yield pheomelanin (Botchkarev, 2003; Bento-Lopes et al., 2023; Orlow et al., 1994; Kobayashi et al., 1998).

### 3.2 Melanosome biogenesis, maturation, and cytoskeletal transport

Melanosome biogenesis progresses through four distinct morphological stages, transitioning from early non-pigmented matrix-containing vesicles (Stages I and II) to progressively melanized structures (Stage III), culminating in fully electron-dense, fully melanized organelles (Stage IV) (D'Alba and Shawkey, 2019; Berson et al., 2001). To coordinate intracellular movement from the perinuclear region to dendritic tips, melanosomes utilize sequential cytoskeletal networks (Fukuda et al., 2021). Long-distance transport along microtubules is mediated by kinesin and dynein motors (Hara et al., 2000), after which melanosomes switch to an actin-based motor complex consisting of myosin Va, Rab27a, and melanophilin (Slac2a) at the cell periphery for local retention (Wu et al., 2001; Provance et al., 2002).

### 3.3 Melanosome transfer and follicular pigmentation

Following peripheral transport, mature melanosomes are transferred from melanocyte dendrites to adjacent epithelial cells, in part *via* filopodia (Bento-Lopes et al., 2023; Scott et al., 2002). While epidermal transfer serves primary photoprotective functions across the skin, follicular pigmentation relies strictly on the transfer of melanosomes into precortical keratinocytes that construct the hair shaft cortex (Tobin, 2011). Protease-activated receptor 2 (PAR-2) and specific cell-surface glycoproteins facilitate this cytocrine process in epithelial tissues (Seiberg et al., 2000). Disruptions in melanosome biogenesis, transport, or transfer manifest clinically as hypopigmentation disorders, such as vitiligo and albinism, or contribute to localized follicular depigmentation during hair graying (Tran et al., 2008; Chang, 2012; Pillaiyar et al., 2018).

### 3.4 Factors regulating follicular pigmentation

Activation and inhibition of melanogenesis are dynamically regulated by intrinsic microenvironmental and extrinsic environmental factors (Serre et al., 2018; D'Mello et al., 2016). Among extrinsic stimuli, ultraviolet (UV) radiation represents a primary driver of epidermal melanogenesis, where UV exposure stimulates melanocyte activation and induces eumelanin production to transform radiation into heat for photoprotection (Serre et al., 2018; d'Ischia et al., 2015). Nonetheless, this classical UV-driven facultative tanning response in the skin is largely non-functional within hair follicle biology (Tobin, 2011). Rather than being directly driven by external radiation, follicular melanogenesis is strictly regulated by localized paracrine and endocrine signaling networks, including WNT, BMP, endothelin, and POMC pathways, that operate within the spatiotemporal framework of the anagen phase of the hair growth cycle (Tobin, 2011). These specific signaling cascades govern melanocyte activation, dictate the enzymatic shift between eumelanin and pheomelanin production, and orchestrate melanosome transfer, thereby determining functional hair shaft pigmentation (Tobin, 2011; Serre et al., 2018).

## 4 Signaling pathways and molecules modulating melanogenesis

Follicular pigmentation is orchestrated by distinct signaling networks that differentially regulate either intrinsic biochemical melanogenesis, including the switch between eumelanin and pheomelanin, or the structural cytocrine transfer of mature melanosomes into precortical matrix cells (Tobin, 2011; Slominski et al., 2005). Figure 3 systematically maps how these upstream paracrine and neuroendocrine cascades converge on MITF within mature anagen bulbar melanocytes to coordinate both enzymatic pigment synthesis within melanosomes and BMP6-assisted melanosome translocation to precortical keratinocytes (Goding and Arnheiter, 2019; Slominski et al., 2005; Singh et al., 2012).

**FIGURE 3:**
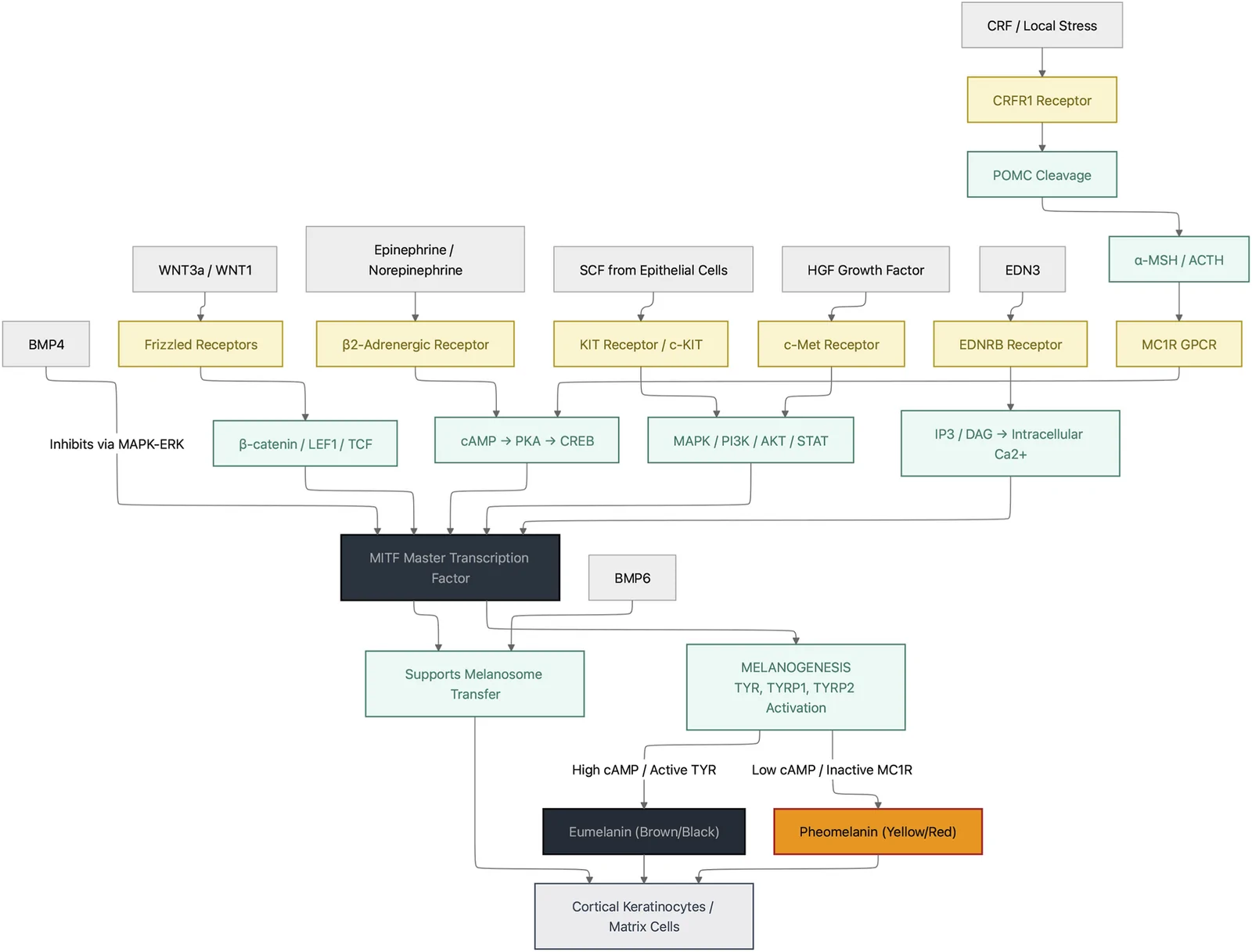
Integrated molecular signaling networks regulating follicular MITF expression and melanosome dynamics. This network diagram maps the autonomous paracrine and neuroendocrine signaling cascades converging onto the master transcription factor MITF within the anagen hair bulb microenvironment. Upstream signals are grouped by functional pathways: canonical WNT/β-catenin signaling, adrenergic stress pathways, receptor tyrosine kinase networks (SCF/KIT and HGF/c-Met), endothelin-3 (EDN3/EDNRB) signaling, and the local neuroendocrine CRF/POMC axis triggering MC1R-mediated cAMP-PKA activation. Solid black arrows indicate positive regulatory signaling that drives downstream transcription, while the T-bar denotes the antagonistic action of BMP4 through MAPK/ERK-mediated MITF phosphorylation. At the bottom of the cascade, divergent paths illustrate how these signaling networks differentially modulate either enzymatic melanogenesis (driving eumelanin *versus* pheomelanin production) or BMP6-supported structural translocation of mature melanosomes into adjacent precortical matrix cells. Signaling networks are mapped according to evidence detailed in (Slominski et al., 2005; Singh et al., 2012; Park et al., 2009; Zhang et al., 2020).

### 4.1 The POMC/MC1R axis and cAMP signaling

In the hair follicle, the pro-opiomelanocortin (POMC) cascade functions as a localized neuroendocrine system independent of external ultraviolet (UV) radiation (Slominski et al., 2005). Intrafollicular cleavage of POMC yields alpha-melanocyte-stimulating hormone (alpha-MSH) and adrenocorticotropic hormone (ACTH) (Nguyen and Fisher, 2019). Binding of alpha-MSH to the melanocortin one receptor (MC1R) on bulbar melanocytes stimulates adenylyl cyclase, elevating intracellular cyclic adenosine monophosphate (cAMP) and activating protein kinase A (PKA) (Wolf Horrell et al., 2016).

This cascade phosphorylates the cAMP response element-binding protein (CREB), directly promoting MITF transcription to upregulate core melanogenic enzymes (TYR, TYRP1, and DCT), thereby specifically promoting dark eumelanin synthesis over pheomelanin (Goding and Arnheiter, 2019). Conversely, when MC1R signaling is impaired or uncoupled, intracellular cAMP levels drop, leading to the default enzymatic shift toward pheomelanin production (Wolf Horrell et al., 2016; Beaumont et al., 2009).

### 4.2 WNT/β-catenin and BMP signaling

The canonical WNT/β-catenin and bone morphogenetic protein (BMP) pathways serve opposing, balanced roles in regulating follicular melanocyte homeostasis. WNT3a signaling stabilizes neural crest-derived progenitors and maintains follicular MITF expression, partnering with lymphoid enhancer-binding factor 1 (LEF-1) to directly drive downstream melanogenic transcription (Yasumoto et al., 2002; Dunn et al., 2005).

Conversely, BMP signaling exhibits divergent functions: BMP4 suppresses enzymatic melanogenesis by inducing acute phosphorylation and degradation of MITF *via* the MAPK/ERK pathway, whereas BMP6 selectively promotes the structural transfer of mature melanosomes into precortical keratinocytes *via* canonical Smad pathways, rather than directly stimulating enzyme expression (Singh et al., 2012; Park et al., 2009)

### 4.3 Endothelin (EDN) signaling pathway

While endothelin-1 (EDN1) mediates UV-induced adaptive tanning in the epidermis, endothelin-3 (EDN3) is the principal isoform orchestrating melanocyte homeostasis and pigmentation within the hair follicle architecture (Mull et al., 2015). Locally synthesized EDN3 binds to endothelin receptor B (EDNRB) on follicular melanocytes, activating phospholipase C (PLC) to generate inositol triphosphate (IP3) and diacylglycerol (DAG), which triggers an influx of intracellular calcium (Stanisz et al., 2012). This calcium influx and downstream MAPKs (ERK1/2 and p38) synergize to phosphorylate CREB and stabilize MITF (Lv et al., 2020). This signaling cascade is indispensable for the maintenance of hair pigmentation; genetic mutations disabling the EDN3/EDNRB axis halt follicular melanocyte differentiation, manifesting as severe coat/hair depigmentation phenotypes across multiple species (Mull et al., 2015).

### 4.4 Tyrosine kinase networks: SCF/KIT and HGF/c-Met

Receptor tyrosine kinase signaling *via* the stem cell factor (SCF)/KIT and hepatocyte growth factor (HGF)/c-Met pathways provides necessary paracrine support for follicular melanocytes. In the hair follicle, SCF is expressed by follicular epithelial cells to activate KIT receptors on bulbar melanocytes, triggering autophosphorylation and sustaining melanogenic gene expression strictly during the anagen phase (Liao et al., 2017). Temporary blockade of KIT signaling induces reversible hair graying without destroying the underlying stem cell niche, proving its cycle-specific requirement (Botchkareva et al., 2001). Similarly, HGF binds to c-Met on follicular melanocytes, activating downstream PI3K/AKT and MAPK/ERK pathways to augment CREB-mediated MITF transcription, thereby co-regulating both follicular melanocyte survival and baseline tyrosinase activity during active hair fiber development (Yu et al., 2015; Czyz, 2018).

### 4.5 Neuroendocrine stress signaling: CRF and adrenergic pathways

The intrafollicular hypothalamic-pituitary-adrenal (HPA) homologue serves as a major mediator of stress-induced hair pigmentation alterations (Slominski et al., 2013). Corticotropin-releasing factor (CRF) and its receptors (CRFR1/2) are highly expressed within the hair follicle unit, where local biological stress upregulates CRF to stimulate intrinsic POMC expression and downstream cAMP-driven melanogenesis (Slominski et al., 2006). However, overactivation of nearby adrenergic signaling disrupts this delicate baseline. Local sympathetic nerves release noradrenaline directly into the bulge niche under acute stress; this sympathetic surge forces quiescent melanocyte stem cells into rapid, abnormal proliferation and differentiation before they can migrate, ultimately exhausting the reservoir and causing irreversible hair graying (Zhang et al., 2020).

## 5 Mechanisms regulating hair graying

To understand the physical consequences of these diverse pathways, physiological hair graying should not be viewed as a sudden, homogeneous event, but rather as a progressive cellular decline spanning distinct follicular compartments across three major phases (Table 1). In the early stage of aging, the emergence of canities is primarily driven by localized oxidative stress and subsequent apoptosis of active, differentiated melanocytes strictly within the hair bulb matrix, while the upper stem cell reservoir remains largely intact (O'Sullivan et al., 2021; Tobin, 2015). As aging advances into the intermediate stage, defects in microenvironmental signaling crosstalk and cell-to-cell adhesion impair the step-wise differentiation and downward migration of progenitor cells along the outer root sheath (ORS) (Nishimura, 2011; Bertrand et al., 2024). Finally, in the late stage, after undergoing multiple hair cycles, the foundational pool of MeSCs within the bulge niche faces permanent depletion or undergoes senescence-coupled differentiation driven by chronic genomic and adrenergic stress (Nishimura et al., 2005; Lv et al., 2020; Mohri et al., 2025). By establishing this chronological framework, the molecular mechanisms detailed below can be structurally mapped to specific spatiotemporal failures within the hair follicle architecture (Figure 4).

**TABLE 1:** Molecular, physiological, and kinetic mechanisms associated with hair graying and cellular senescence.

Mechanisms	Pigmented hair follicles	Hypo-pigmented hair follicles	References
Melanogenic and enzymatic activities			
Enzyme activities of TYR, TYRP1, and TYRP2	Active expressions	Abrupt downregulation at catagen	(Van Neste and Tobin, 2004)
Sizes of melanosomes and the Golgi apparatus	Normal Stage IV melanosomes and Golgi	Fewer, smaller, and vacuolated degradation	(Van Neste and Tobin, 2004)
Follicular growth and structural kinetics			
Growth speed of hair follicles	Slow linear growth speed	Accelerated growth rate and larger diameter	(Tobin, 2015; Van Neste and Tobin, 2004)
KRT and KRTAP expressions	Baseline hard hair keratin matrix	Ectopic over-expression of KRT6/14/16/25	(Jo et al., 2018)
Upstream growth factor balance	Balanced FGF5 and FGF7 levels	Reduced FGF5 and increased FGF7	(Jo et al., 2018)
Stem cell niche and cellular adhesion			
Stem cell niche maintenance	Stable reservoir and high BCL2 levels	Shifting balance and loss of BCL2 with age	(Nishimura et al., 2005)
Cellular migration and adhesion	Intact Dicer-miR92b signaling	Defective cascade and loss of alpha V integrin	(Bertrand et al., 2024)
Genotoxic stress checkpoint	Intact DNA repair mechanisms	p53-p21 pathway and seno-differentiation	(Mohri et al., 2025)
Oxidative stress and microenvironment			
Oxidative stress	Low ROS and efficient baseline clearance	Destructive ROS and uncoupled oxidation	(O'Sullivan et al., 2021; Tobin, 2015; Ji et al., 2017)
Hydrogen peroxide	Normal clearance *via* active catalase	H2O2 accumulation	(Seiberg, 2013)
Antioxidative enzymes	Normal	Depleted catalase and SOD	(Seiberg, 2013)
Norepinephrine signaling	Tonic sympathetic balance preserving niche	Hyperactivation and norepinephrine surge	(Zhang et al., 2020)
Niche sensitivity to radiation	Resilient MeSCs self-renewal	Acute HFSC epithelial dysfunction	(Aoki et al., 2013)

**FIGURE 4:**
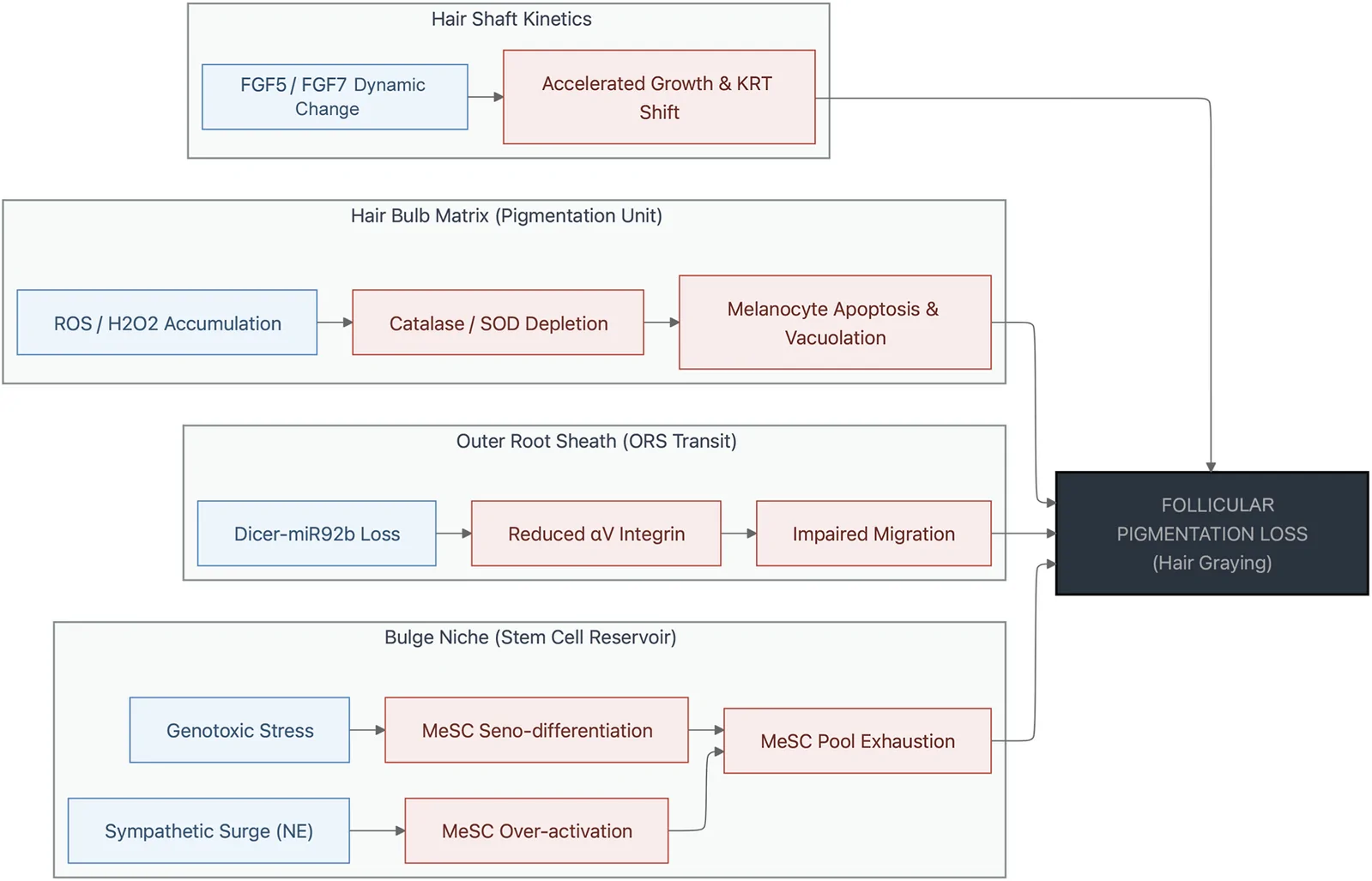
Schematic overview of extrinsic and intrinsic factors driving compartment-specific dysfunction during hair graying. This schematic diagram illustrates how multifactorial stress inputs selectively target distinct cellular compartments within the hair follicle architecture to induce canities. Sympathetic overactivation releasing noradrenaline and genotoxic stress force premature proliferation and senescence-coupled differentiation (seno-differentiation) of melanocyte stem cells (MeSCs) within the bulge niche. Disruption of the Dicer-miR92b pathway impairs integrin-mediated progenitor migration along the outer root sheath. Within the anagen hair bulb, reactive oxygen species (ROS) and hydrogen peroxide accumulation trigger vacuolation and apoptosis of mature, pigment-producing melanocytes. Concurrently, upstream growth factor alterations (reduced FGF5 and increased FGF7) alter hair shaft structural kinetics, accelerating linear growth rate and hard keratin expression in unpigmented follicles.

### 5.1 Aging-related hair graying

Hair and skin appearances are dynamically modulated by nutritional, psychoemotional, and oxidative statuses, alongside fundamental variables such as ancestral background, biological sex, and chronological age. Among these, aging-related hair graying occurs prominently across nearly all populations (Tobin, 2015). This process is highly linked to systemic endocrine shifts; for instance, sex hormones including androgen and estrogen fluctuate during puberty and senescence, significantly impacting long-term follicular pigmentation capacity (Poonia et al., 2024).

Intriguingly, the onset of physiological hair graying exhibits notable variations across diverse genetic lineages. Onset typically begins earliest in individuals of European ancestry (at 34 ± 9.6 years of age), followed by those of Asian ancestry, and appears latest in individuals of African ancestry (at 43.9 ± 10.3 years) (Kumar et al., 2018). Furthermore, the spatial distribution of graying varies by sex; hair graying in men usually initiates in the occipital and temporal regions before spreading toward the vertex, whereas women display a more diffuse pattern across the scalp (Panhard et al., 2012).

### 5.2 Premature hair graying

Premature hair graying represents an accelerated onset of canities in young individuals, though its precise chronological definition varies across distinct ethnic subgroups. Genetically, premature hair graying is generally defined as appearing before the age of 20 years in individuals of European ancestry, 25 years in those of Asian ancestry, and 30 years in those of African ancestry (Acer et al., 2019; Kaur et al., 2019). Because hair graying is strongly associated with senescence, premature hair graying frequently triggers significant psychological distress and reduced self-esteem in affected patients.

The precise etiology of premature hair graying remains multi-factorial, driven by interactions between genetic predisposition, oxidative stress, and nutritional deficiencies, such as vitamin B12 depletion or chemotherapeutic interventions (Kaur et al., 2019). Mechanistically, chronic oxidative stress in the hair follicle disrupts the Dicer–miR92b–ItgαV pathway; a downregulation of Dicer impairs microRNA 92b (miR92b), which subsequently reduces downstream αV integrin (ItgαV) levels, causing severe melanocyte migratory defects and cell death (Bertrand et al., 2024). This structural damage progressively diminishes the melanocyte stem cell (MeSC) pool within the bulge niche, resulting in an irreversible depletion of active melanogenesis (Acer et al., 2020). Mechanistically, Mohri et al. revealed that severe genotoxic stress forces MeSCs into antagonistic fate decisions; rather than transforming, damaged MeSCs activate the p53-p21 pathway to undergo cellular senescence-coupled differentiation (seno-differentiation). This permanent depletion results in hair graying, which essentially functions as an evolutionary safeguard that eliminates potentially malignant clones to protect against melanoma development (Mohri et al., 2025).

### 5.3 Stem cell niche environment

Follicular aging is inherently coupled with decreased stem cell regenerative capacity and a shrinking progenitor pool (Oh et al., 2014). In the human scalp, the hair follicle pigmentation machinery functions efficiently for only about 10 hair cycles (Van Neste and Tobin, 2004). Throughout these cycles, usually up to the age of 45 years, MeSCs must continually proliferate and differentiate to replenish the outer root sheath and hair bulb cohorts roughly 7 to 15 times (Van Neste and Tobin, 2004).

Maintaining a strict balance within this microenvironment is essential to prevent premature depletion. The localized reservoir of MeSCs within the hair follicle bulge decreases progressively with age, a process tightly modulated by anti-apoptotic factors like BCL2 (Nishimura et al., 2005). Additionally, this niche is regulated by neighboring hair follicle stem cells through coordinated cell-to-cell signaling, ensuring synchronized activation alongside the hair cycle (O'Sullivan et al., 2021; Tanimura et al., 2011).

### 5.4 Oxidative stress

An age-related imbalance alters tissues’ oxidative stress and, in turn, affects the MeSC and melanocyte homeostasis. The melanocyte state is dynamically altered along the hair growth cycle phases: melanocytes activate and differentiate in anagen, undergo apoptosis in catagen, and are quiescent in telogen (Oh et al., 2016). In human scalp hair follicles, pigmentation activity of bulbar melanocytes can last for 10 years (Van Neste and Tobin, 2004). Reactive oxygen species (ROS) are produced in large quantities through the oxidation of TYR or DOPA to form melanin (O'Sullivan et al., 2021). Melanin acts as both a free radical scavenger and a potential oxidant in melanogenesis (Brenner and Hearing, 2008; Sikkink et al., 2020) (Figure 1). On the other hand, pheomelanin enhances the formation of ROS (Morgan et al., 2013). However, a direct correlation between this pheomelanin-driven ROS burden and an accelerated timing of premature hair graying, for instance in individuals with red hair, remains unconfirmed due to a lack of direct evidence.

External factors, such as hair treatment processing, smoking, and UV exposure, may also lead to ROS accumulation and, in turn, reduce the antioxidant capacity. Such ROS accumulation causes nuclear and mitochondrial DNA damage, raising mutational and pathological risks (such as inflammation, melanoma, and aging), while restraining the proliferation and transformation of both melanocytes and the hair bulb epithelium (O'Sullivan et al., 2021; Tobin, 2015; Ji et al., 2017; Rosenberg et al., 2021).

Hydrogen peroxide accumulation and antioxidative enzyme depletion are phenomena that can be detected in aging melanocytes and follicular melanocytes. Expression and activity levels of catalase in follicular melanocytes decrease more than those in the epidermis during aging. The increase in hydrogen peroxide accumulation occurs due to an imbalance of activation of superoxide dismutase (SOD) and a reduction in catalase, consequently enhancing the aging phenotype and affecting the innate antioxidant system of gray hair bulbar melanocytes (Seiberg, 2013).

Melanin can function both as an antioxidant and a potential source of oxidative stress. Under physiological conditions, melanogenesis is inherently oxidative; the enzymatic oxidation of tyrosine to dopaquinone naturally generates intracellular ROS as metabolic byproducts. Furthermore, melanin polymers serve critical physiological functions as broad-spectrum free radical scavengers and photoprotective agents. Under pathological conditions, however, ROS become destructive only when an age-related uncoupling occurs, specifically characterized by a sharp decline in protective antioxidant enzymes (catalase and SOD) coupled with the accumulation of hydrogen peroxide. This tipping point shifts ROS from physiological signaling intermediates into pathological drivers of nuclear and mitochondrial DNA damage, lipid peroxidation, and cellular senescence.

### 5.5 Norepinephrine (NA) and β2 adrenergic pathway

Norepinephrine (NA), a stress hormone, is a major contributor to stress-induced hair graying. NA accelerates this process by binding to β2 adrenergic receptors (β2AR) on MeSCs, driven by the hyperactivation of sympathetic nerves. This interaction shifts MeSCs from quiescence to proliferation, leading to their rapid differentiation and depletion (Zhang et al., 2020). NA signaling further disrupts melanogenesis by affecting intracellular calcium balance and promoting ectopic cellular differentiation (Li et al., 2023; Rachmin et al., 2021). Crucially, a quantitative mapping study by Rosenberg et al. demonstrates that stress-induced canities is not a strictly unidirectional senescent trajectory during normal hair growth; rather, hair graying operates as a dynamic, reversible process where spontaneous repigmentation can occur across individual hair shafts, a reversal phenomenon tightly coupled with the mitigation of life stress (Rosenberg et al., 2021).

### 5.6 Transformation of the organelles, melanosomes and Golgi apparatuses

The overall appearance of gray hair is usually a mix of pigmented and unpigmented hairs. During aging, individual hair follicles lose their pigment for several growth cycles, which leads to an unpigmented hair shaft. To interpret this more thoroughly, there is an evident reduction of melanosomes in cortical keratinocytes of senile hair shafts. Melanocyte degeneration causes defective melanosome transfer and melanin production in graying hair bulbs and the neighboring epidermis. Furthermore, non-melanogenic melanocytes fail to express the functional receptor binding sites for the pro-eumelanogenic peptide, α-MSH (Van Neste and Tobin, 2004) (Figure 3). Concurrently, hair follicle innervation and localized neuropeptide signaling significantly modulate the melanocyte stem cell niche and the progression of canities (Zhang et al., 2020; Chen et al., 2022). The microscopic morphology of melanocytes changes when melanogenic activity decreases, and organelles (including melanosomes and Golgi apparatuses) in remaining melanocytes become fewer and smaller. In order to deal with increased oxidative stress, melanocytes are commonly highly vacuolated in gray hair bulbs. Consequently, autophagolysosomal degradation of melanosomes occurs after melanocyte degeneration (Van Neste and Tobin, 2004).

### 5.7 Ionizing radiation

Beyond chronological aging, ionizing radiation represents a potent extrinsic trigger for hair graying. While it was initially assumed that MeSCs were the primary direct targets of radiolytic damage, comparative evidence indicates a niche-mediated mechanism. Following radiation exposure, MeSCs maintain relatively stable self-renewal activity; conversely, neighboring hair follicle stem cells (HFSCs) exhibit high sensitivity to radiation-induced damage (Aoki et al., 2013). These findings highlight a microenvironmental mechanism where radiation-induced hair graying is primarily driven by the acute dysfunction of surrounding follicular epithelial cells, which fail to sustain the active pigmentation unit, rather than intrinsic defects within the melanocyte lineage itself (Aoki et al., 2013).

### 5.8 Melanogenic enzyme activity

During the progression of chronological hair graying, the enzymatic machinery of the follicular pigmentation unit undergoes severe functional impairment. Studies demonstrate that bulbar melanocytes in graying hair follicles exhibit a marked, progressive downregulation of key melanogenic enzymes, including tyrosinase (TYR), tyrosinase-related protein 1 (TYRP1), and dopachrome tautomerase (DCT/TYRP2) (Van Neste and Tobin, 2004). This loss of enzymatic activity is closely coupled with reduced upstream MITF transcription and overwhelming oxidative stress, which together compromise both DOPA oxidase capacity and melanosome maturation, ultimately resulting in the production of unpigmented hair shafts (O'Sullivan et al., 2021; Paus et al., 2024).

### 5.9 Secondary structural and kinetic divergence between pigmented and unpigmented follicles

Beyond optical depigmentation, the deactivation of the follicular pigmentation unit induces secondary structural and kinetic alterations, leading pigmented and unpigmented hair follicles to display markedly distinct growth profiles. Clinically, senile unpigmented hair follicles exhibit a significantly accelerated linear growth rate and a larger hair shaft diameter compared to their pigmented counterparts within the same scalp microenvironment (Tobin, 2015; Van Neste and Tobin, 2004). This kinetic disparity suggests a distinct metabolic trade-off within the hair bulb architecture; the massive amount of cellular energy, tyrosine substrates, and nutrients normally consumed by active biochemical melanogenesis and structural melanosome transfer may be fully redirected toward cortical keratinocyte proliferation and hair fiber extension once the follicular pigmentation unit is deactivated (Tobin et al., 2005).

This kinetic acceleration is consistently accompanied by a major transcriptional shift within the hair shaft structural matrix, reflecting altered differentiation programs in the follicular epithelium. Unpigmented hair follicles exhibit a more extensive expression of specific hard keratins, including KRT6, KRT14/16, and KRT25 (Jo et al., 2018). This structural alteration is coordinately regulated by upstream follicular growth factors; specifically, the expression levels of fibroblast growth factor 5 (FGF5) are significantly suppressed, whereas FGF7 levels are boosted in unpigmented follicles, acting as the primary drivers modulating downstream keratin and keratin-associated protein (KRTAP) gene expressions to accommodate the accelerated hair growth speed (Jo et al., 2018) (Figure 4).

### 5.10 A model of hair graying

Integrating the molecular, cellular, and kinetic findings discussed above, here we propose a novel, three-stage chronological model of hair graying to conceptualize how distinct follicular compartments functionally decline over time: (1) early stage: localized accumulation of ROS and depletion of catalase in the hair bulb overwhelm the antioxidant capacity of mature, differentiated melanocytes. This triggers localized apoptosis of active pigment-producing units without immediately destroying the upper progenitor pool; (2) intermediate stage: as chronological aging or stress persists, defects emerge in microenvironmental signaling (such as WNT/β-catenin and SCF/KIT downregulation) and cell adhesion pathways (e.g., the Dicer-miR92b-ItgαV axis), impeding the step-wise differentiation and downward migration of progenitor cells along the outer root sheath; (3) late stage: after repeated hair cycles, the foundational reserve of MeSCs undergoes permanent depletion or p53-p21-driven seno-differentiation under chronic genotoxic and sympathetic stress, resulting in irreversible canities.

### 5.11 Differences between mouse and human melanocytes

Mice and humans share core cellular mechanisms of hair graying, primarily the depletion and functional exhaustion of MeSCs, yet significant translational divergence exists between murine experimental paradigms and human clinical phenotypes due to distinct hair-cycling dynamics and architectural longevity (O'Sullivan et al., 2021; Van Neste, 2004). In mice, synchronized and rapid hair cycling enables acute experimental manipulations; consequently, key mechanistic models, such as acute sympathetic hyperactivation driving noradrenaline-mediated MeSC burst-proliferation and niche depletion (Lv et al., 2020), or lineage-traced genotoxic stress triggering p53-p21-driven seno-differentiation (Mohri et al., 2025), have been established predominantly in murine systems. In contrast, human scalp hair follicles display asynchronous cycling with prolonged anagen phases lasting several years, operating within a lifelong cumulative exposure to microenvironmental stress. Classical histopathological findings in human canities demonstrate progressive catalase depletion, extensive mitochondrial vacuolation, and an age-associated reduction of the bulge reservoir over decades (O'Sullivan et al., 2021 Van Neste, 2004).

Crucially, recent single-cell RNA sequencing and spatial transcriptomic investigations have provided unprecedented resolution to bridge this cross-species gap (Wu et al., 2022). Comparative single-cell mapping between pigmented and senile unpigmented human hair follicles from identical individuals demonstrates that human hair graying may not be solely governed by the premature total loss of the stem cell pool, but rather by an arrest in lineage progression, characterized by a marked depletion of matrix transit-amplifying cells (TACs) and localized p53 pathway activation (Wu et al., 2022). Furthermore, spatial profiling reveals localized DNA damage, epithelial senescence signatures, and microenvironmental immune cell infiltration surrounding graying human hair bulbs (Wu et al., 2022). These emerging spatial and single-cell datasets emphasize that while mouse models provide indispensable mechanistic blueprints of acute stem cell fate decisions, human canities represents a multifaceted tissue-level decline where localized microenvironmental niche degeneration and regenerative arrest interact to drive progressive depigmentation.

## 6 Medical conditions causing hair repigmentation

Hair graying is a common change during human aging, and generally non-reversible. However, a few clinical reports have documented cases of repigmentation under specific medical conditions or treatments (Yale et al., 2020; Chan et al., 2019; Rivera et al., 2017; Shaffrali et al., 2002; Rosenberg et al., 2021; Tintle et al., 2015). Of note, hair repigmentation is not a universal side effect of these medical conditions or medications; therefore, the connections between them still require further investigation. Although the exact mechanisms of most of these clinical cases are unclear, it is important to determine whether hair repigmentation is directly caused by reactivation of follicular melanocytes. If this is the case, melanocytes may enter a transiently inactive state within aged gray or white hair follicles, which would allow the possibility of future approaches to reactivate melanocytes in gray/white hair follicles. In the following, we summarize several rare clinical hair repigmentation cases that were associated with specific clinical conditions or treatments. Again, these are rare cases representing a very small population of patients in the groups.

### 6.1 Programmed cell death (PD)-1 and PD ligand 1 (PD-L1) immunotherapy

A clinical report showed 14 cases of hair repigmentation in 52 patients with lung cancer. These patients had received anti-PD-1 (11 received nivolumab and one received pembrolizumab) or anti-PD-L1 (two received atezolizumab) therapy (Rivera et al., 2017). The mechanism mediating this phenomenon remains unclear, but we think it is likely associated with immunomodulation during anti-PD1/PD-L1 therapy. Hair follicles possess immune privilege due to factors like TGF-β and low MHC I expression. Inflammation may disrupt this privilege, activating melanocytes through paracrine signaling. PD-1/PD-L1 immunotherapy may induce such inflammation, leading to hair follicle activation and potential repigmentation (Manson et al., 2018; Feng et al., 2023). On the other hand, the expression of PD-L1 in qMeSCs may be the mechanism of repigmentation. The clinical presentation of the PD-1/PD-L1 immunotherapy can support the hypothesis that PD-L1-positive qMeSCs persist within unpigmented and gray hair. Although PD-L1 appears dispensable for normal hair repigmentation *in vivo*, the study predicts that PD-L1-positive qMeSCs could become therapeutic targets for hair-graying reversal under specific conditions (Palmer et al., 2024).

While recent experimental lineage models indicate that a distinct subset of PD-L1-expressing qMeSCs resists age-related depletion by restricting cell cycling, direct evidence confirming their functional role in human hair graying reversal is currently lacking. We now emphasize that while PD-L1 marks an important reservoir of stress-resistant stem cells in experimental models, translating this mechanism into targeted human therapeutics remains a speculative hypothesis requiring extensive clinical validation.

### 6.2 ROS scavengers

Oxidative stress is seen as the dominant reason that leads to aging-related hair graying. A study focused on the target of the reduction-oxidation reaction (redox) balance to reverse hair graying. The “smart active ingredient” was composed of glucosylated taxifolin (dihydroquercetin, DHQ) and epigallocatechin gallate (EGCG). These molecules possess ROS-scavenging properties, which can reduce ROS levels and, in turn, maintain the redox balance. The ability to modulate MeSC apoptosis was also demonstrated. Clinically, hair pigmentation occurred after only 4 months of twice-daily application (De Tollenaere et al., 2021). In conclusion, these ingredients can trigger a repigmentation process, protect melanocyte reservoirs in hair follicles, and inhibit canities through regulating several melanogenesis-related genes and transcription factors (De Tollenaere et al., 2021).

### 6.3 Tumor necrosis factor (TNF) inhibitors

TNF inhibitors are widely used medications for several inflammatory diseases such as psoriasis, rheumatoid arthritis, and inflammatory bowel disease, and hair loss is a rather general side effect of these medical therapies compared to hair color change. It was reported that a patient had hair repigmentation after adalimumab therapy. Adalimumab, a fully human monoclonal immunoglobulin G1 (IgG1) antibody, binds to both soluble and transmembrane TNF. TNF is prominently known to inhibit melanogenesis. It is supposed that TNF restrains the melanosome transfer in the microenvironment of hair follicles (Fu et al., 2020; Wang et al., 2013). As a result, the patient had hair color change from grayish-white to brownish-black over the frontal, temporal, lateral parietal, and occipital regions of the scalp (Tintle et al., 2015).

### 6.4 Melanomas

Melanomas are a cancerous disease that arises from highly proliferative melanocytes due to genetic mutations. Melanomas are not usually associated with a change in hair color. However, several clinical cases reported hair repigmentation caused by a melanoma *in situ* of the scalp (Chan et al., 2019; Tiger et al., 2014; Inzinger et al., 2013). These observations show the ability of neoplastic melanocytes to replace or activate the lost function of follicular melanocytes. It is not clear whether these melanoma cells directly contribute melanin to hair follicle keratinocytes or they reactivate follicular melanocytes *via* paracrine signaling, such as SCF/KIT (Figure 3). Of note, in these clinical cases, the repigmented hairs are usually lighter than their original hair color (Chan et al., 2019).

### 6.5 Porphyria cutanea tarda

Porphyria cutanea tarda is a rare skin lesion with painful blistering caused by sun exposure. Clinical cases present darkened hairs after a diagnosis of porphyria cutanea tarda (Shaffrali et al., 2002). Although the mechanism remains elusive, this phenomenon is possibly a post-inflammatory effect of inflamed tissues.

### 6.6 Mechanism-based pharmacological strategies

Beyond disease-associated repigmentation, recent experimental models have proposed mechanism-based pharmacological interventions to prevent or reverse canities. *In vitro* and animal studies indicate that the selective serotonin reuptake inhibitor, fluoxetine, enhances local follicular expression of alpha-MSH to stimulate melanogenesis (Liao et al., 2012), whereas the p53 inhibitor drug, pifithrin-alpha, protects MeSCs from genotoxic stress-induced depletion (Mohri et al., 2025), highlighting targets for future topical therapeutic research.

A recent single-cell RNA-sequencing study of human scalp hair follicles mapped their lineage trajectories and discovered that hair graying is not caused by stem cell loss, but rather by a specific reduction of matrix transit-amplifying cells and the activation of the P53 signaling pathway. By comparing black and gray hair follicles from the same individuals, researchers found that gray follicles retain stem cells while showing DNA damage and immune infiltration. Furthermore, experiments using a mouse model demonstrated that applying the P53 inhibitor pifithrin-alpha can successfully suppress matrix cell depletion and ameliorate hair graying (Wu et al., 2022).

## 7 Conclusion

Hair graying is a hallmark of human aging; however, the exact mechanism causing hair hypopigmentation during aging remains unclear. Melanocytic activity can be regulated by both intrinsic and extrinsic factors (Serre et al., 2018). In this review, we discussed the physiology and pathologies of the melanin biosynthesis pathway, melanocyte differentiation and activation, molecules and pathways associated with melanocytes and their microenvironment, and hair pigmentation and hypopigmentation.

Laying the foundation of the microenvironment may be the first step toward repigmentation, due to the importance of the hair follicle microenvironment in affecting the development of melanocytes. For example, avoiding psychological stress and potential physical damage to hair follicles can be beneficial for maintaining the function of the pigmentation unit. Currently, the most common method for managing graying hair is the use of temporary hair dye. Physical therapies, such as photodynamic therapy and microneedling, can serve as alternative approaches for repigmentation. Overall, understanding the intricate molecular signaling networks governing follicular melanogenesis provides a rational framework for future translational strategies.
